# Neonatal survival and determinants of mortality in Aroresa district, Southern Ethiopia: a prospective cohort study

**DOI:** 10.1186/s12887-019-1907-7

**Published:** 2020-01-27

**Authors:** Alaka Adiso Limaso, Mesay Hailu Dangisso, Desalegn Tsegaw Hibstu

**Affiliations:** 1 School of Public Health, Hawassa, Ethiopia; 2 School of Public Health, College of Medicine and Health Sciences, Hawassa University, Hawassa, Ethiopia

**Keywords:** Neonatal survival, Determinants, Mortality, Aroresa, Southern Ethiopia, Prospective cohort

## Abstract

**Background:**

The first 28 days of aliveness are the biggest challenge mentioned for the continuity of life for children. In Ethiopia, despite a significant reduction in under-five mortality during the last 15 years, neonatal mortality remains a public health problem accounting for 47% of under-five mortality. Understanding neonatal survival and risk factors for neonatal mortality could help devising tailored interventions. The aim of this study was to determine the neonatal survival and risk factors for neonatal mortality in Aroresa district, Southern Ethiopia.

**Methods:**

A community based prospective follow up study was conducted among a cohort of term pregnant mothers and neonates delivered from January 1/2018 to March 30/2018. A total of 586 term pregnant mothers were selected with a multistage sampling technique and 584 neonates were followed-up for a total of 28 days, with 12 twin pairs. Data were coded, entered cleaned and analyzed using SPSS version 22. Kaplan–Meier survival curve was used to show pattern of neonatal death in 28 days. Independent and adjusted relationships of different predictors with neonates’ survival were assessed with Cox regression model. The risk of mortality was explored and presented with hazard ratio and 95% confidence interval and *P*-value less than 0.05 were considered as significant.

**Result:**

The overall neonatal mortality was 41 per 1000 live births. Hazards of neonatal mortality was high for neonates with complications (AHR = 3.643; 95% CI, 1.36–9.77), male neonates (AHR = 2.71; 95% CI, 1.03–7.09), neonates that mothers perceived to be small (AHR = 3.46; 95% CI, 1.119–10.704), neonates who had initiated exclusive breast feeding (EBF) after 1 h (AHR = 3.572; 95% CI, 1.255–10.165) and mothers who had no postnatal care (AHR = 3.07; 95% CI, 1.16–8.12).

**Conclusion:**

Neonatal mortality in the study area was 4.1% which was high and immediate action should be taken towards achieving the Sustainable Development Goals. To improve neonatal survival, high impact interventions such as promotion of maternal service utilization, essential newborn care and early initiation of exclusive breast feeding were recommended.

## Background

Neonatal mortality (NNM) is the death of a baby within the first 28 days of life and is expressed as neonatal deaths per 1000 live births. The first 28 days of aliveness are the biggest challenge mentioned for the continuity of life for children. Figures in 2015 pointed out that nearly six million under- five died before celebrating their fifth year; close to one million newborns will lose their life at the first day of their birth. A one million newborns will die in the early neonatal period and, about 2.8 million will die in the late neonatal period though the overall neonatal mortality rate went down by 49% from 37 in 1990 to 19 deaths per 1000 live births in 2016 [1].

Neonatal mortality has become an important public health issue in many developing countries. Among newborns in sub-Saharan Africa, about 1 among 36 children dies in the neonatal period, while in the world’s richest countries the neonatal death is 1 in 333 children [1]. Africa is one of the global regions that show the smallest declines in Neonatal Mortality Rate (NMR) in the so far Millennium Development Goal. In Sub-Saharan Africa, neonatal mortality accounts for 35% of all child deaths [2]. Ethiopia is the third highest neonatal mortality contributor in Africa with 187,000 neonatal deaths in 2015 [3].

According to Ethiopian Demographic Health Survey (EDHS) 2016, the neonatal mortality rate in Ethiopia was 29 deaths per 1000 live births [4]. Leading causes for neonatal death are pre-term birth, severe infections, and asphyxia. Neonatal factors like birth size, birth rank and birth interval and maternal complication during labour, as well as health service seeking behavior are the potential determinants of neonatal mortality [5].

The Ethiopian government has formulated and implemented a number of policies including Integrated Management of Newborn and Childhood Illness (IMNCI) strategy [6], Kangaroo Mother Care (KMC) [7] and Health Sector Development Plan (HSDP) [8], which aim at continued improvements in childhood survival by increasing access to and quality of health services to every segment of the society. Despite these policy and intervention initiatives, currently, Ethiopia has the third highest reported number of newborn deaths in Africa and ranks fifth having the highest number of deaths globally [1] and unluckily, the available information on neonatal mortality is overly scarce to blueprint locally specific interventions [1]. Therefore, this study was aimed at determining determinants of neonatal survival in Aroresa District, Southern Ethiopia.

## Methods

### Study setting and period

The study was conducted in Aroresa district which is one of 23 districts in Sidama Zone, Southern Nations, Nationalities and Peoples’ Region (SNNPR), Ethiopia. It is located at the distance of 181 kms from Hawassa, the capital of SNNPR and 554 kms from Addis Ababa. The district has 30 rural and 3 urban *Kebeles* (the smallest administrative unit in Ethiopia) with a total population of 220,332 and of this, females constitute 49.8%. The women of reproductive age group account for 51,337(23.3%) of the total population. According to the district health office report, the total number of estimated deliveries in 2017/18 was 7623 and proportion of the utilization of first ANC, institutional delivery, Postnatal care (PNC) services and contraceptive prevalence were 77, 38, 69 and 49%, respectively. The district has one primary hospital, 8 health centers, 33 health posts, 3 private clinics and 4 private drug stores [Aroresa District Health Office, Aroressa District annual plan, unpublished]. The study was conducted from January 1/2018 to March 30/2018.

### Study design and population

A community based prospective cohort study was conducted among a cohort of term pregnant mothers and neonates delivered from January 1/2018 and March 30/2018 in randomly selected kebeles. All term pregnant mothers (≥37 week Gestational Age (GA)) who live in the study kebeles were included in this study and followed up until they gave birth and their neonates were followed-up for a total of 28 days. All term pregnant mothers who had a known psychiatric disorder, unable to speak and residents for less than 6 months were excluded from this study.

### Sample size and sampling procedure

The sample size was calculated using single population proportion with the assumption that the proportion of neonatal mortality was 2.9% [4], margin of error 2%, confidence interval of 95%, and a design effect (DE) of 2. Using the sample size correction formula and adding 15% non-response and lost to follow-up rate, and the final sample size became 600.

Multistage sampling technique was used to identify 600 term pregnant women to be enrolled in the follow up for the study (all term pregnant mothers were recruited consecutively until sample size was reached). First, all the *Kebeles*: in Aroresa district were determined to be 33. Then, 10 *kebeles* (9 rural and 1 urban) were selected from the district by simple random sampling method using OpenEpi 3.03. The calculated sample size was proportionally allocated to each study kebele based on expected number of term pregnant women per ‘*Kebele*’. Then the calculated sample was selected consecutively from each *kebele*.

### Variables

The outcome variable is neonatal survival dichotomized as (alive =1 and died = 0) The predictor variables include; *socio-demographic* and *economic factors:* ethnicity, religion, place of residence, marital status, education status of mother and father, and occupational status of mother, age of mother, *maternal factors*: age at child birth, maternal complication (excessive bleeding, puerperal sepsis and fever, prolonged labour, eclampsia and preeclampsia malpresentation and malposition, premature rupture of membrane, and obstructed labour), *maternal service utilization factors*: place of delivery, ANC visit, postnatal care, initiation of exclusive breast feeding (EBF) and *neonatal factors*: birth size, birth order and interval and neonatal complication like asphyxia, infection, hypothermia, and jaundice.

### Operational definition

*Neonatal death***:** a death of neonate within 28 days of life according to report of mother participated in the study. *Neonatal survival* is defined as being alive up to the end of follow-up period (28 days). *Term pregnancy* is a pregnancy between 37 completed weeks up to 42 completed weeks of gestation*. Birth size* is defined as the size of newborn at birth according to the perception of mother. *Stillbirth* is defined as any fetus born without a heartbeat, respiratory effort or movement, or any other signs of life. *Neonatal complication* in this study is defined as a neonate experiencing at least one or more of the following conditions; asphyxia, hypothermia, jaundice, convulsion, unable to breast feed or any conditions which endangers the life the neonate. According to maternal estimate, mother’s perception of baby’s size is defined in this study as small equivalent to less than 2500 g, average 2500 g to 4000 g and large greater than 4000 g. This estimate was obtained because birth weight is unknown for most (86%) newborns in Ethiopia however the mother’s estimate of weight is subjective and interpretation of the finding should be viewed with caution [4].

### Data collection tool and procedure

A structured questionnaire, first prepared in English and translated into Sidamu Afoo (local language), were employed to collect the data. All term (> 37 week GA) pregnant women at selected kebeles were identified by Health Extension Workers (HEW). Trained data collectors were contacted the women to obtain informed consent, to perform interviews and later to conduct postpartum follow-up, home visits, at week 1, and 4. All data collectors were contacted with the supervisor by mobile phone every week and on site supervision. Baseline data collected during recruitment were maternal socio-demographic information, medical history and use of health services like antenatal care. Pregnancy outcomes, the circumstances of delivery, date of birth, date of death of neonate, feeding patterns, and illness episodes of neonate were collected during follow-up period. The data collection processes were supervised strictly by trained supervisors and the principal investigator.

The quality of data was assured using a properly designed questionnaire, proper training of the interviewers and supervisors about the data collection and follow-up procedures, proper categorization and coding of the questionnaire. Questionnaires were pre-tested on 5% of the sample outside the study area. Data were double entered and screened for missing, outlier values and data entry errors using the frequency distribution. Errors were corrected against the raw data and the necessary corrections were made before the analysis.

### Statistical analysis

Data were coded, entered, cleaned and analysed using SPSS version 22. Pregnancy outcome variables were explained by descriptive statistics and neonatal outcome variables were examined against all confounding variables using regression analysis. Kaplan–Meier survival curve was used to show pattern of neonatal death in 28 days. Independent and adjusted relationships of different predictors with neonates’ survival were assessed with Cox regression model. The risk of mortality was explored and presented with hazard ratio and 95% confidence interval. *P*-value less than 0.05 was considered as significant. Multicollinearity between the independent variable was assessed using variance inflation factors (VIF) and VIF greater than 10 was considered as existence of multicollinearity before interpreting the final output.

## Results

A total of 586 term pregnant mothers were identified and enrolled in follow up consecutively for 3 months period and these pregnancies resulted in 14 (2.4%) stillbirths and 584 live births, including 12 pairs of twins. Then 584 neonates were followed until 28 days to determine their survival status. From a total of 600 neonates planned, 584 neonates were recruited making a response rate of 97.33%. The perinatal mortality (stillbirth and early neonatal deaths) was 49/1000 pregnancies.

The mean age of mothers was 29.08 years [+ 4.37 standard deviation (SD)], 523(89.6%) mothers were residing in rural areas at the time of delivery of their last child and 553(94.7%) were married. Regarding educational status, 231(39.6%) of mothers were illiterate and 156(28.2%) of husbands had attended primary school. The median household’s monthly income was 550 Ethiopian Birr (ETB) or 18.71 US dollar (1US dollar = 29.40 ETB), [Interquartile Range (IQR) =537.5] and 420 (72.1%) of mothers were housewives (Table 1**).**

**Table 1:** Socio demographic characteristics of mothers and neonates in Aroresa district, Southern Ethiopia, 2018

Variables	Categories	Frequency (n.)	Percentage (%)
Age of mother	15–24	117	20.0
	25–34	418	71.6
	> 34	49	8.4
Place of residence	Urban	61	10.4
	Rural	523	89.6
Marital status	Married	553	94.7
	Others^a^	31	5.3
Husband educational level	Cannot read and write	151	27.3
	Read and write	145	26.2
	Primary	156	28.2
	Secondary & above	101	18.3
Maternal educational level	Illiterate	231	39.6
	Read and write	147	25.2
	Elementary	105	18.0
	Secondary & above	101	17.3
Religion	Protestant	520	89.0
	Orthodox	23	3.9
	Catholic	7	1.2
	Muslim	34	5.8
Ethnicity	Sidama	513	87.8
	Amhara	39	6.7
	Oromo	12	2.1
	Guragie	20	3.4
Maternal occupation	Government employee	30	5.1
	Merchant	93	15.9
	Housewife	420	71.9
	Others^b^	41	7.0
Monthly income (ETB)	< 300	90	15.4
	301–500	201	34.4
	501–1000	186	31.8
	> 1000	107	18.3

^a^single, divorced, widowed ^b^ Student, daily labor

### Characteristics of term pregnant mothers and their neonates

Among 584 neonates born in the study area, 405 (69.3%) born from mothers who had ANC follow up and, of whom 209 (35.8%) had 1–2 ANC visits in health facilities. Five hundred and fifty seven (95.4%) of mothers had no history of chronic medical diseases and 500 (85.6%) had no history of previous still births. Majority of neonates, 501 (85.8%) born from mothers who had no complication during delivery. More than half, 318 (54.5%) of neonates born in home and 520 (89.0%) had started breast feeding within 1 h.

Concerning the characteristics of neonates, 300 (51.4%) were females, 294 (50.5%) were in 3rd and 4th birth order or birth rank and 560 (95.9%) were singleton in birth type. From 584 neonates, 422 (72.3%) had no neonatal complication during neonatal period and 52.9% had postnatal care during their neonatal period **(**Table 2).

**Table 2:** Characteristics of mothers and neonates in Aroresa district, Southern Ethiopia, 2018

Variables	Category	Frequency (n)	Percentage
Birth order	1st and 2nd	108	18.5
	3rd and 4th	294	50.5
	5th and above	182	31.2
History of still birth	Yes	84	14.4
	No	500	85.6
History of chronic diagnosed medical illness	Yes	27	4.6
	No	557	95.4
ANC visit	Yes	405	69.3
	No	179	30.7
Number of ANC visits	None	179	30.7
	1–2	209	35.8
	3 and 4	196	33.6
Complication during labor	Yes	83	14.2
	No	501	85.8
Place of deliver	Health facility	266	45.5
	Home	318	54.5
Baby sex	Boys	284	48.6
	Girls	300	51.4
Birth type	Single	560	95.9
	Twin	24	4.1
Baby’s size	Average	404	69.2
	Small	60	10.3
	Large	120	20.5
Birth interval	No previous birth	61	10.4
	< 24 months	233	39.9
	24–48 months	258	44.2
	> 48 months	32	5.5
Initiation of breast feeding	< 60 min	520	89.0
	> 60 min	64	11.0
Neonatal complication	Yes	162	27.7
	No	422	72.3
Post natal care	Yes	309	52.9
	No	275	47.1

According to the perception of mothers on size of their neonates, 404 (69.18%) reported average size and 258 (44.2%) were 24–48 months in birth interval.

### Neonatal survival

Among 584 neonates followed, there were 24 neonatal deaths making a neonatal mortality rate (NMR) of 41 per 1000 live births (95% CI: 26–58). Over all, the neonatal survival was 95.9% (95% CI: 94.2–97.4). From 24 neonatal deaths, 15 (62.5%) deaths occurred in the first week, 5 (20.83%) occurred at the second week, 3 (12.5%) occurred at third week and 1 (4.17%) occurred at last week.

The incidence rate of neonatal mortality was 1.51 per 1000 person-days-of observation.

### Kaplan-Meir survival analysis

**Fig. 1:**
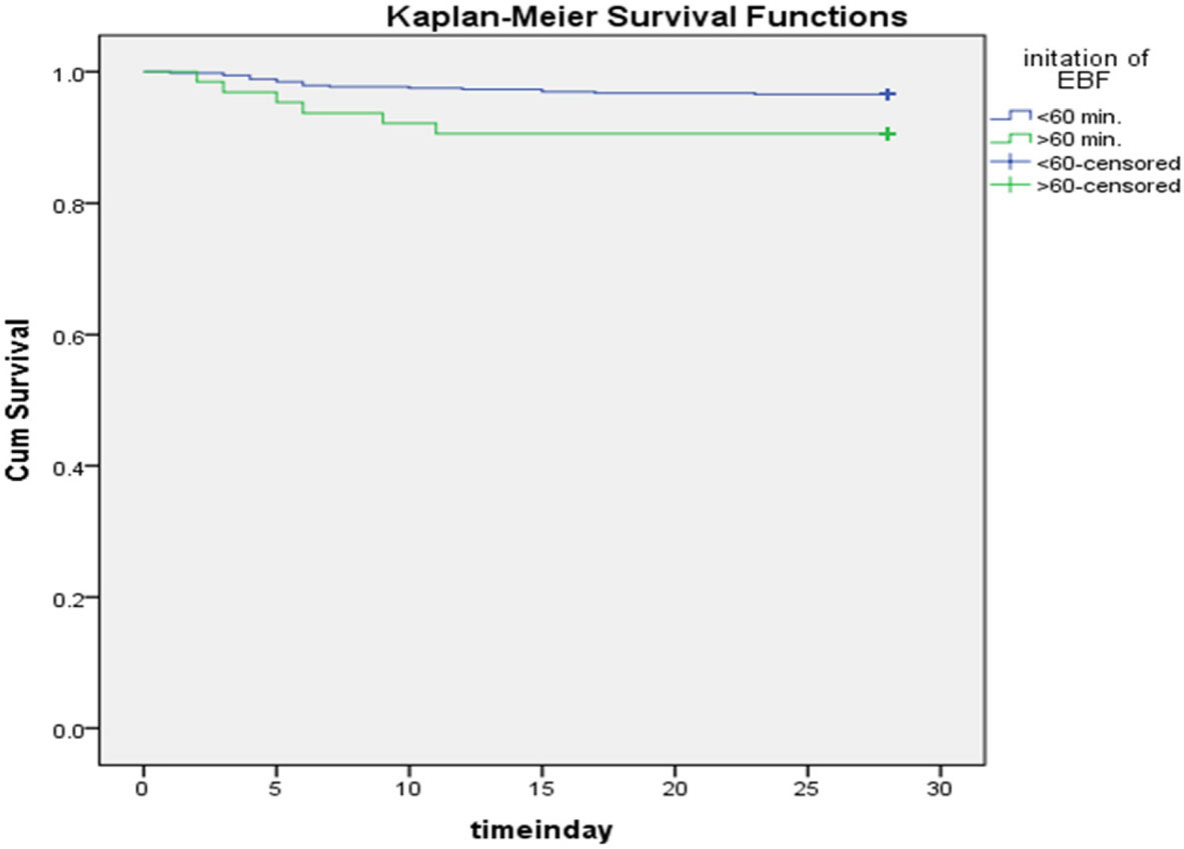
Kaplan-Meir survival pattern of Neonate and initiation of Exclusive Breast Feeding (EBF) Aroresa district, Southern Ethiopia, 2018

Kaplan-Meier survival function curve shows that the probability of survival of a neonate who had initiated EBF after 1 h was lower than a neonate who had initiated EBF within 1 h. On day 5, the probability of survival of a neonate who had initiated EBF after 1 h was 95.3% and for a neonate who had initiated within 1 h was 98.5% (Fig. 1). Moreover survival graph indicates that the probability of survival of both groups falls rapidly on first week and looks stable after the end of second week.

### Cox proportional hazards regression models

Variables with p- value less than 0.25 in crude model were included in the Cox proportional hazards regression model. Maternal educational status, neonatal complication, maternal history of stillbirth, place of child birth, baby’s sex, baby’s size at birth, birth type, initiation of BF and postnatal care were variables included in Cox regression model. Maternal history of stillbirth, place of child birth and birth type lost their significance after adjusting for confounders.

The risk of neonatal mortality was about 3.6 times higher for neonates who had neonatal complication compared to those who had no complication (AHR = 3.64, 95% CI 1.39–9.77). Male neonates had 2.7 times higher risk of neonatal mortality compared with female neonates (AHR 2.71, 95% CI 1.04–7.09). According to mother’s perception of baby’s size, neonates that mothers perceived to be small had 3.46 times more risk of dying compared with average size neonates (AHR 3.46, 95% CI 1.119–10.704). Time of initiation of BF and postnatal care were also independent predictors of neonatal mortality. Neonates initiated BF after 1 h and who had no postnatal care were about 3.6 (AHR = 3.57, 95% CI 1.26–10.17)**)** and 3 (AHR = 3.07, 95% CI 1.16–8.12) times at higher risk of dying than neonates initiated BF within 1 h and had postnatal care, respectively **(**Table 3**)**.

**Table 3:** Determinants of Neonatal Survival in Aroresa district, Southern Ethiopia, 2018

Variables	Neonatal Survival Status		CHR (95% CI)	AHR (95% CI)	*p*-value
	Censored N (%)	Died N (%)			
Maternal educational status					
Illiterate	215(93.1)	16(6.9)	7.18(0.95–54.13)	2.45(0.31, 19.60)	0.399
Read and write	142(96.6)	5(3.4)	3.44(0.40–29.46)	3.43 (0.39, 29.97	0.265
Primary	98(98.0)	2(2.0)	1.94(0.18–21.37)	1.45(0.13, 16.35	0.77
Secondary and above	105(99.1)	1(0.9)	1.00	1.00	
Neonatal complication					
Yes	146(90.1)	16(9.9)	5.39(2.31–12.59)	3.64 (1.36, 9.77)	0.010^*^
No	414(98.1)	8(1.9)	1.00	1.00	
History of still birth					
Yes	260(97.7)	6(2.3)	2.55(1.01–6.42)	1.13(0.41, 3.10	0.810
No	300(94.3)	18(5.7)	1.00	1.00	
Place of deliver					
Health facility	260(97.7)	6(2.3)	1.00	1.00	
Home	300(94.3)	18(5.7)	2.55(1.01–6.42)	2.00(0.67, 5.97	0.214
Baby sex					
Male	267(94.0)	17(6.0)	2.61 (1.08–6.29)	2.71(1.04, 7.09	0.042^*^
Female	293(97.7)	7(2.3)	1.00	1.00	
Birth type					
Single	539(96.3)	21(3.8)	1.00	1.00	
Twin	21(87.5)	3(12.5)	3.48(1.04–11.68)	3.99 (0.96, 16.63)	0.057
Baby’s size					
Average	393(97.3)	11(2.7)	1.00	1.00	
Small	51(85.0)	9(15.0)	5.89(2.44–14.22)	3.46(1.12, 10.70)	0.031^*^
Large	116(96.7)	4(3.3)	1.24(0.40–3.90)	1.37(0.41,4.55)	0.609
Initiation of breast feeding					
< 60 min.	502(96.5)	18(3.5)	1.00	1.00	
> 60 min.	58(90.6)	6(9.4)	2.81(1.12–7.09)	3.57(1.26, 10.17)	0.017^*^
Postnatal care					
Yes	302(97.7)	7(2.3)	1.00	1.00	
No	258(93.8)	17(6.2)	2.799(1.16–6.75)	3.07(1.16, 8.12)	0.024^*^

^*^ = significant at *P* < 0.05

## Discussion

Our study showed that stillbirth rate in the study area was 24 per 1000 births which is much lower than hospital based prospective cohort study in Uganda which reported stillbirth rate of 120 per 1000 births [9]. This finding is also lower than EDHS 2016 report of perinatal mortality but it is higher than the study conducted in South-west Ethiopia [10]. Our result should be interpreted cautiously because it reports the stillbirth rate among term pregnancy only, which might underestimate actual burden of the stillbirth in the study area. On the other hand, there could be a misclassification of pregnancy outcome such as; severely asphyxiated neonates might be classified as stillbirth which could also overestimate the magnitude of stillbirth rate in the study setting.

The neonatal mortality rate in the study area was 4.1%, which was higher than the global neonatal mortality rate in 2016 [1] and reports from studies conducted in Ethiopia [11, 12], Sudan and Zambia [13, 14] . The differences might be attributed to study designs, health service coverage and socio-economic factors. Our result is consistent with the NMR of Pakistan [1], studies conducted in Jimma, Ethiopia and Nigeria [15, 16], but it is lower than a study done in Tigray, Ethiopia [17]. In our study, 62.5% neonatal deaths occurred in the first week of their life, which is similar with other studies [11, 17, 18]. The higher mortality in the study setting could be due poor access to quality maternal and child care services, high proportion of home deliveries, poor access to skilled attendants, where the coverage of skilled birth attendants is 28% in Ethiopia and Southern Ethiopia [4], and particularly midwives in rural settings. Tailored and high impact maternal and child care interventions should be implemented to avert avoidable deaths.

Regarding determinants of neonatal survival, socio demographic factors contribute to neonatal mortality in this study. Additionally, place of child birth also contributes less to neonatal mortality, even though it was reported as one of the strongest predictors of neonatal mortality in different studies [15, 19, 20].

Our study revealed that male neonates were less likely to survive than female neonates. This is similar with studies done in different areas even though there are differences in the strength of association [18, 21–24]. In this study male neonates were about 2.7 times more likely to die than female neonates. This finding is consistent with the study conducted in Northern Shoa, Ethiopia and higher than studies conducted in Nigeria, Pakistan, Bangladesh and Indonesia [18, 23–24]. The possible explanations for the increase in mortality among male neonates, as mentioned in many studies, could be male sex appeared to have respiratory problems, intrauterine growth restriction insufficiency or prematurity and low Apgar score [25, 26].

We did not measure the birth weight of newborns since the study was conducted at community level; instead we used mother’s perception of baby’s size to estimate the size of neonates. Our result shows that neonates that mothers perceived to be small were 3.46 times at risk of dying than those with average in size. Similarly, a study from Indonesia revealed that newborns whose birth size according to mothers’ perception was smaller than average, had 2.8 times higher risk of dying than average sized babies and neonates of low birth weight had 5.5 times higher risk of neonatal death than normal weight babies [23]. Additionally, findings from Nigeria shows that neonates perceived as small or smaller by their mothers were 2.26 times at higher risk of dying than average sized neonates [21]. Another study from Pakistan also reported that a hazard of neonatal mortality was higher among small sized neonates than average sized neonates [22]. The prospective follow up study in Jimma, Ethiopia also showed that small size babies at birth were found to have an increased risk of death than average size neonates [15].

Post-natal care in this study was statistically significant determinant of neonatal survival. Neonates who had no post natal care during neonatal period had 3 times higher risk of neonatal mortality than neonates who had post natal care. A study from Ghana consistently reported that utilization of post natal service was inversely related with neonatal mortality [27].

Similarly, the study conducted in Northern Shoa, Ethiopia revealed that neonates born from mothers who did not receive postnatal service were 3 times more likely to experience neonatal death than neonates who born from mothers who received postnatal services [19]. This might be due to postnatal visits which could enable the HEWs to identify and screen health condition of mothers and their neonates and this could help in providing neonatal health care services. Our finding had a lower strength of association compared with a study conducted in Jimma, Ethiopia. The study reported that neonates having poor neonatal care had about ten times higher risk of dying during neonatal period as compared to those who received good comprehensive neonatal care [15]. A possible explanation for this difference might be due to classification of study variables. The studies classified postnatal care as having poor comprehensive neonatal care and good comprehensive neonatal care unlike our study.

In this study, neonates initiated breast feeding after 1 h of delivery were at higher risk of dying during neonatal period. This agrees with the studies conducted in other parts of Ethiopia. The case control study in North Shoa Zone, Ethiopia indicated that neonatal death was in excess among neonates who were not breastfed within the first hour after delivery compared with those who were breastfed within the first hour after delivery [19]. A study from Tigray, Ethiopia also reported initiating exclusive breast feeding within 1 h has a protective effect on hazards of neonatal mortality [19]. Initiation of breast feeding within the first hour can help prevent neonatal deaths caused by infections such as sepsis, pneumonia, and diarrhea and may also prevent additional hypothermia related deaths, especially in preterm and low birth weight infants in developing countries [28].

Our study showed that neonates with complication survive less likely than neonates who had no complications. The study from Tigray, Ethiopia reported consistently that neonate who had no complications after birth had 99.86% less hazard of death than who had complications [17]. Similarly, the study from Ghana revealed that infections, preterm birth and low birth weight, birth trauma, and hypothermia were causes of neonatal mortality [29]. Preterm birth complications, intra partum related events and sepsis or meningitis were reported as among the leading causes of newborn deaths globally in 2016 [30]. In our study, preterm pregnant mothers were not participated in the study which might underestimate the strength of association of neonatal complication and neonatal mortality.

As limitations, in this study, baby weight was not measured but estimated from mother’s perception of baby’s size which was subjective and may cause information bias. However, measures were taken to minimize the bias by helping mothers correctly estimate the size of their babies. The results are also consistent with other studies in using similar methods. We included term pregnant mothers and their newborns. This could affect or underestimates the neonatal and perinatal mortality rates. However, the study provided valuable information about the magnitude of the problem in the study setting. The study did not address all determinants of neonatal mortality which might affect true association of studied variables and unmeasured factors.

## Conclusion

Neonatal mortality in study area was still high and calls for immediate action towards achieving the Ethiopian Health Sector Transformation plan of reducing neonatal mortality to 10% at the end of 2020 and the targets of the Sustainable Development Goals. A significant proportion of mothers also delivered at home, which requires strategies to improve coverage of institutional delivery. Various factors such as neonatal complications, duration of EBF, sex of neonates, size of neonates at birth, and postnatal care were identified as independent predictors of neonatal survival. Tailored interventions addressing modifiable risk factors should be devised so as to improve the neonatal survival in the study area.

## Data Availability

The data that support the findings of this study will be found from the representing author on reasonable request in the form of SPSS.

## Abbreviations

AHR: Adjusted Hazard Ratio
ANC: Antenatal Care
CHR: Crude Hazard Ratio
CI: Confidence Interval
DE: Design Effect
EBF: Exclusive Breast Feeding
EDHS: Ethiopian Demographic and Health Survey
GA: Gestational Age
HEW: Health Extension Worker
HSDP: Health Sector Developmental Program
IMNCI: Integrated Management of Newborn and Childhood Illness
IRB: Institutional Review Board
KMC: Kangaroo Mother Care
NMR: Neonatal Mortality Rate
SPSS: Statistical Package for Social Sciences

## References

[1] IGME U. Levels & Trends in Child Mortality: Report 2017, Estimates Developed by the UN Inter-Agency Group for Child Mortality Estimation. United Nations Children’s Fund, New York. 2017.

[2] UNITED NATIONS (2015). The millennium development goals report.

[3] UNICEF (2015). Committing to Child Survival: A Promise Renewed Progress Report 2015.

[4] Central statistical Agency (CSA)[Ethiopia] and ICF (2016). Ethiopia Demographic and Health Survey 2016.

[5] Fort AL, Kothari MT, Abderrahim N (2008). Association between maternal, birth and newborn characteristics and neonatal mortality in five Asian countries. DHS working papers no. 55.

[6] Federal Minister of Health/MCH Directorate (2015). National Newborn and Child Survival Strategy Document Brief Summary 2015/16–2019/20.

[7] Save the childern (2017). Kangaroo mother care in Ethiopia.

[8] Federal Ministry of Health, Ethiopia (2015). Health Sector Development Program IV 2010/11–2014/15 FINAL DRAFT.

[9] Nakimuli A, Mbalinda SN, Nabirye RC, Kakaire O, Nakubulwa S, Osinde MO, Kakande N, Kaye DK (2015). Still births, neonatal deaths and neonatal near miss cases attributable to severe obstetric complications: a prospective cohort study in two referral hospitals in Uganda. BMC Pediatr.

[10] Yaya Y, Eide KT, Lindtjørn B, Norheim OF (2014). Maternal and neonatal mortality in south-West Ethiopia: estimates and socio-economic inequality. PLoS One.

[11] Desta BN, Assefa N, Damte TD, Hordofa LO (2016). Neonatal Mortality and its risk factors in Eastern Ethiopia: A Prospective Cohort Study in Kersa Health and Demographic Surveillance System (Kersa HDSS). Mortality.

[12] Shifa GT, Ahmed AA, Yalew AW (2016). Early days of life are crucial for child survival in Gamo Gofa zone, southern Ethiopia: a community based study. BMC Pediatr.

[13] Bashir AO, Ibrahim GH, Bashier IA, Adam I (2013). Neonatal mortality in Sudan: analysis of the Sudan household survey, 2010. BMC Public Health.

[14] Lukonga E, Michelo C. Factors associated with neonatal mortality in the general population: evidence from the 2007 Zambia demographic and health survey (ZDHS); a cross sectional study. Pan Afr Med J. 2015;20(1). 10.11604/pamj.2015.20.64.5616 PMC4450022 26090022

[15] Debelew Gurmesa Tura, Afework Mesganaw Fantahun, Yalew Alemayehu Worku (2014). Determinants and Causes of Neonatal Mortality in Jimma Zone, Southwest Ethiopia: A Multilevel Analysis of Prospective Follow Up Study. PLoS ONE.

[16] Akinyemi JO, Bamgboye EA, Ayeni O (2015). Trends in neonatal mortality in Nigeria and effects of bio-demographic and maternal characteristics. BMC Pediatr.

[17] Mengesha HG, Wuneh AD, Lerebo WT, Tekle TH (2016). Survival of neonates and predictors of their mortality in Tigray region, Northern Ethiopia: prospective cohort study. BMC Pregnancy Childbirth.

[18] Yared M, Biruk T, Daniel ST, Tedbabe D, Abeba B (2013). Neonatal mortality in Ethiopia: trends and determinants. BMC Public Health.

[19] Kolola T, Ekubay M, Tesfa E, Morka W (2016). Determinants of neonatal mortality in north Shoa zone, Amhara regional state, Ethiopia. PloS one.

[20] Abdullah A, Hort K, Butu Y, Simpson L (2016). Risk factors associated with neonatal deaths: a matched case–control study in Indonesia. Glob Health Action.

[21] Ezeh OK, Agho KE, Dibley MJ, Hall J, Page AN (2014). Determinants of neonatal mortality in Nigeria: evidence from the 2008 demographic and health survey. BMC Public Health.

[22] Nisar YB, Dibley MJ (2014). Determinants of neonatal mortality in Pakistan: secondary analysis of Pakistan demographic and health survey 2006–07. BMC Public Health.

[23] Titaley CR, Dibley MJ, Agho K, Roberts CL, Hall AJ (2008). Determinants of neonatal mortality in Indonesia. BMC Public Health.

[24] Mondal MN, Hossain MK, Ali MK (2009). Factors influencing infant and child mortality: a case study of Rajshahi District, Bangladesh. J Human Ecol.

[25] Aibar L, Puertas A, Valverde M, Carrillo MP, Montoya F (2012). Fetal sex and perinatal outcomes. J Perinat Med.

[26] Hou L (2014). Cross sectional study in China: fetal gender has adverse perinatal outcomes in mainland China. BMC Pregnancy Childbirth.

[27] Kayode GA, Ansah E, Agyepong IA, Amoakoh-Coleman M, Grobbee DE, Klipstein-Grobusch K (2014). Individual and community determinants of neonatal mortality in Ghana: a multilevel analysis. BMC Pregnancy Childbirth.

[28] Begum K, Dewey KG. Impact of early initiation of exclusive breastfeeding on newborn deaths. 2010.

[29] Diedrich CM (2016). Neonatal mortality in Ghana.

[30] MCEE W. MCEE-WHO methods and data sources for child causes of death 2000–2015. World Health Organization; 2016.

